# Comparison of General-Social Health and Corona-Induced Anxiety Between Active and Inactive Students in the COVID-19 Pandemic

**DOI:** 10.3389/fpsyt.2021.798947

**Published:** 2021-12-21

**Authors:** Somayeh Ahmadabadi

**Affiliations:** Department of Physical Education and Sport Sciences, Farhangian University, Tehran, Iran

**Keywords:** anxiety, COVID-19, exercise, health, social health

## Abstract

**Background:** General health includes physical and mental health and their interactions, and physical activity can improve people's mental and social health. The present study has compared general-social health and COVID-19-induced anxiety between active and inactive students during the COVID-19 pandemic.

**Methods:** A causal-comparative research design was used in this study. The statistical population consisted of all students of the Farhangian University of Mashhad (2,500 students) in 2020, out of whom 752 students were randomly selected (323 men and 429 women). Subjects were assessed for general health, social health, and COVID-19-induced anxiety. Data normality was investigated by the Kolmogorov-Smirnov test and data were analyzed statistically using independent *t*-test and Pearson correlation coefficient test.

**Results:** The results of the present study showed that there was a significant difference between general health and COVID-19-induced anxiety of active and inactive students (*p* = 0.001), but no significant difference was observed between these two groups in social health (*p* ≥ 0.05). Results obtained regarding the correlation indicated that there was a significant correlation between general and social health in both active and inactive students (*p* = 0.001). However, there was no significant correlation between COVID-19-induced anxiety and general-social health (*p* ≥ 0.05).

**Conclusion:** According to the results of the present study, it can be said that an active lifestyle and physical activity are important factors to improve general health and reduce anxiety, especially in specific conditions of the COVID-19 outbreak. Therefore, it is recommended that students have a regular exercise program to reduce their anxiety and increase the level of their physical health.

## Introduction

The COVID-19 outbreak around the world has led to some health measures (such as the reduced use of sports and recreational facilities and traffic in such places as parks and playgrounds, self-quarantine for people who may be infected with the virus, social distancing and avoiding social gatherings, and limited contact with the elderly and people with poor health) to reduce the spread of the virus and restrict unnecessary traffic (1). Therefore, due to these health measures, people are expected to change their behavior, lifestyle, and the level of their physical activities in daily life. While the priority has been given to protect people from disease as much as possible, the side effects of health measures can reduce physical activity and increase sedentary or inactive lifestyle, which put society at greater risk and lay the grounds for further deterioration of chronic diseases (2). Additionally, the challenges of increasing the level of physical activity in public places of recreation and the different behaviors of people in such places can be due to individual, social, and psychological differences.

Thus, despite the strong advice of public health advocates to increase the level of physical activity in the home, the destructive effects of quarantine on lifestyle can be seen. A significant increase in psychological effects of the coronavirus epidemic and the quarantine such as stress, confusion, and anger has been reported by the research (3). Other psychological and social effects include social isolation, financial insecurity, job loss, and childcare challenges. Social health is the ability to perform effectively and efficiently social roles without harming others. It is associated with social skills, social performance, and the ability to recognize oneself as a member of a larger community (4). However, the effects have not only been limited to psychological problems and the disease has also led to poverty and social welfare problems (5), physical health problems and heart disease (6), educational problems (7), and nutritional problems (8). As the level of physical activity is strongly correlated with mental and general health, research has shown that active people tend to experience less stress, depression, and anxiety than their peers (9). That is why regular physical activity has been considered in recent years as a potential treatment for depression and anxiety in addition to medications or instead of them (10). Additionally, research has shown an increase in the benefits of physical activity outdoors with increased exposure to nature. The positive psychological effects of exposure to nature include a sense of happiness, improved mood, self-esteem, vitality, and reduced stress (11).

So far, most studies have been related to psychological problems caused by coronavirus disease in patients, their families, and medical personnel. In this regard, Feng et al. (12) examined anxiety and depression in 148 patients with coronavirus disease using a self-report scale. The rate of anxiety was reported to be 21.63% and the rate of depression was 50%. Additionally, studies conducted on the psychological conditions of medical personnel during the epidemic coronavirus have shown that the average scores of psychological disorders, including anxiety and fear, are significantly higher in these people compared to other people in the community (13). On the other hand, studies conducted on the general public have also shown that psychological problems have increased during the coronavirus pandemic. Ripon et al. (14) showed that the symptoms of post-traumatic stress disorder and depression increased more, especially in low-income people, during the quarantine period than in the previous times. As the various dimensions of the psychological and social consequences of coronavirus disease are still unknown, the variables that can exacerbate or weaken them are not yet fully understood. Therefore, further research is needed to understand the effects of an active lifestyle on the quality of general-social health and anxiety in different people when facing such events as the coronavirus pandemic. Accordingly, the present study compares general-social health and anxiety among active and inactive students during the COVID-19 pandemic.

## Materials and Methods

A causal-comparative research design was used in this study. The statistical population consisted of all students of the Farhangian University of Mashhad in two campuses (2,500 students) in 2020 including 1,000 women and 1,500 men. After obtaining the necessary permissions from the university officials, the online questionnaire link (in the context of Porsline) was provided to students through WhatsApp and Telegram as tools used by the university for the students' online learning. The students were asked to answer all the questions carefully. The online questionnaire consisted of the section of demographic information, General Health Questionnaire (GHQ-28), Keyes Social Health Questionnaire, and Corona Disease Anxiety Scale (CDAS). At the beginning of the questionnaire, a complete explanation of the research purpose was provided and the students answered the questions after documenting their informed consent to participate in the research. A telephone number and email address were provided to students for any possible questions. One thousand three hundred ninety four students entered the survey system, 752 people (323 men and 429 women) answered the questionnaire completely and registered it.

Out of whom, 329 people (37% men, 63% women) were active students.

In this study, the criterion for distinguishing active and inactive students was their self-report of weekly physical activity so that active students had engaged in regular moderate- and vigorous-intensity physical activity for 1 h on 3 days per week during the past year. The selected active students all met these conditions.

The remaining 423 subjects were inactive students (45% men, 55% women). The subjects were compared in terms of general health, social health, and COVID-19-induced anxiety using the General Health Questionnaire (GHQ-28), Keyes Social Health Questionnaire, and Corona Disease Anxiety Scale (CDAS). This study received the approval of the research ethics committee of Mashhad University of Medical Sciences with a code of IR.MUMS.REC.1399.445.

General Health Questionnaire (GHQ-28): it is one of the most popular tools developed to assess non-psychotic psychiatric disorders in the general population. This screening tool has been designed to identify short-term changes in general health and focuses on two main concerns. The first one is the inability to carry out normal functions and the second one is the appearance of new phenomena that lead to anxiety. In the present study, the 28-item general health questionnaire was used, which has been designed by Goldberg (1979) (15). The questions of this questionnaire examine an individual's mental state in the last month and cover four main areas of somatic symptoms, anxiety, insomnia, social dysfunction, and severe depression. The psychometric properties of the Persian version of this questionnaire were examined by Taghavi (16). It was recognized that the questionnaire enjoyed good psychometric properties so that its test-retest reliability was equal to 0.70 and its internal consistency, obtained using Cronbach's alpha, was equal to 0.90.

Keyes Social Health Questionnaire: This questionnaire was used to collect data. It consists of 32 questions that assess five components of social health (social cohesion, social prosperity, social acceptance, social participation, and social adaptation). Social prosperity means believing that society is growing positively. This component includes seven questions. Social cohesion means feeling should be a part of society, belong to it, be supported by it, and have a share in it. This component also includes seven questions. Social acceptance means having a positive attitude toward people, acknowledging others, and generally accepting people despite some of their confusing and complex behaviors. This component includes six questions. Social participation means feeling that people have something valuable to offer to society and thinking that their daily activities are valued by society. This component also includes six questions. The last component, social adaptation, indicates to believe that society is understandable, logical, and predictable, to know and be interested in society and its concepts. This component includes six questions (17). To investigate the reliability and validity of the Social Health Questionnaire, Sharbatiyan (18) conducted a study on the students of Mashhad University of Medical Sciences in 2012 and obtained Cronbach's alpha of 0.90 for the variable of social health. In this study, social health was assessed by the score obtained by each participant in the Keyes Social Health Questionnaire.

Corona Disease Anxiety Scale (CDAS): This tool was developed by Alipour et al. (19) in 2019 to measure anxiety caused by the outbreak of the coronavirus in Iran. Based on Cronbach's alpha, good validity and reliability were reported for mental symptoms (α = 0.879), somatic symptoms (α = 0.861) and the whole questionnaire (α = 0.919). The final version of this tool includes 18 items. The items measure mental and somatic symptoms and are scored using a 4-point Likert scale (never = 0, sometimes = 1, very often = 2, and always = 3); therefore, the highest and lowest scores that the respondents get in this questionnaire are 0 and 54. High scores indicate a higher level of anxiety in individuals. The reliability of this tool (obtained using Cronbach's alpha) has been equal to 0.919. It consists of questions related to the rate of searching for news about the coronavirus pandemic, the infection of close relatives to coronavirus, the death of close relatives due to coronavirus disease, the anxiety caused by coronavirus disease, and the communication conflicts in the family during the quarantine (19).

In the present study, the normal distribution of data was investigated by the Kolmogorov-Smirnov test and the data were analyzed statistically using independent *t*-test and Pearson correlation coefficient test. The software used was SPSS 21 and the significance level was determined to be 0.05.

## Results

The results obtained from the Kolmogorov-Smirnov test demonstrated the normal distribution of data. Table 1 presents the demographic features of the study population. Out of 752 people who participated in this study, 323 subjects were men and 429 subjects were women. In terms related to marital status, 611 subjects were single and 141 subjects were married. The age range of the participants was between 18 and 22 years and there was no significant difference between the two groups of active and inactive students in terms of demographic features (Table 1).

**Table 1:** Descriptive indicators of research variables by the two groups of active and inactive students.

**Number**	**Active students**	**Inactive students**
N	329	423
Age	20.3 ± 2.15	20.34 ± 31.51
Height	171.99 ± 4.89	168.04 ± 9.49
Weight	60.91 ± 11.79	63.90 ± 15.68
BMI	21.41 ± 2.64	22.10 ± 4.82
General health	31.48 ± 18.11	25.36 ± 14.02
Social health	60.92 ± 4.89	60.73 ± 5.63
Corona anxiety	21.81 ± 1.67	22.54 ± 3.13
History of infection	(%9.03) 30	(%9.3) 39
Death of first-degree relatives caused by corona	(%4.2)14	(%2.9)12

Table 2 presents the results obtained from the independent *t*-test to determine the differences between subjects in the variables of general health, social health, and COVID-19- induced anxiety (Table 2).

**Table 2:** The results obtained from the independent *t*-test regarding the differences between subjects in the two groups of active and inactive students.

**Variable**	**Group**	**M ± SD**	**Mean difference**	**df**	***t***	***P***
General health	Active	31.48 ± 18.11	6.119	750	140.266	0.001^*^
	Inactive	25.36 ± 14.02				
Social health	Active	60.92 ± 4.89	0.191	750	0.397	0.692
	Inactive	22.54 ± 3.13				
Corona anxiety	Active	21.81 ± 1.67	−0.721	750	−0.85	0.004^*^
	Inactive	22.54 ± 3.13				

* *Significance at the level of P < 0.05*.

In Table 3, the results obtained regarding the correlation between the research variables in the two groups of active and inactive students show that there was a significant correlation between general health and social health of active and inactive students during the coronavirus pandemic. However, no significant correlation was observed between COVID-19- induced anxiety, general health, and social health (*p* ≥ 0.05) (Table 3).

**Table 3:** Results obtained regarding the correlation between the research variables in the two groups of active and inactive students.

**Group**	**Variable**	**General health**		**Social health**	
		***r***	***P***	***R***	***P***
Active	Corona anxiety	0.021	0.785	0.009	0.905
Inactive		0.041	0.319	0.010	0.818
Active	Social health	0.156	0.044^*^	-	-
Inactive		0.377	0.001^*^	-	-

* *Significance at the level of P < 0.05*.

## Discussion

Teachers have a very significant impact on students, their health should be evaluated. Student teachers, as prospective teachers, need to have good general-social health. Therefore, the focus of this study was to investigate general-social health of active and inactive student teachers in the COVID-19 pandemic. The results of the present study indicated that there was a significant difference between the two groups of active and inactive students in terms of general health and COVID-19- induced anxiety. However, there was no significant difference between the two groups in social health. Correlation results showed that there was a significant correlation between general health and social health in both groups of active and inactive students, but no significant correlation could be seen between COVID-19- induced anxiety and general-social health.

Lesser and Nienhuis (20) examined the impact of COVID-19 on the physical activity behavior and well-being of Canadians. In this study, it was observed that public health measures considered due to coronavirus disease had made changes in the physical activity of people and the access to related places such as recreation facilities, national parks, and playgrounds in Canada. While the public health priority is to keep Canadians safe as much as possible, the unintended consequences may be a reduction in physical activity and an increase in sedentary behavior, exposing the population to an increased risk and an opportunity for deterioration in chronic health conditions (2). On the other hand, to examine the rate of physical activity in different situations, it is necessary to understand the determining factors that affect people's engagement in physical activity. These factors include individual psychological factors, social environment, and finally physical environment including access to low-cost recreation facilities and outdoor physical activity opportunities (19). Physical activity in different ways can lead to the improved level of the general-social health of people in the community. According to Sonstorme's psychological model, engaging in various physical activities can lead to increased self-esteem in individuals. It is obvious that increased self-esteem can bring about positive changes in interpersonal relationships and social networks. Such changes can help to improve the general-social health of people. The development of social relationships, either directly through making new friends or indirectly by increasing self-esteem and establishing interpersonal relationships based on communication patterns, increases a person's courage in the face of stressful events (9).

The results demonstrated no significant difference between the two groups in social health. It can be related to the conditions of the students of Farhangian University. Because these students, from the beginning of their study in the university, are employed as prospective teachers and receive a monthly income. They enjoy guaranteed employment and income, and in terms of social conditions, they have good conditions compared to students of other universities. Therefore, as social and economic status, and employment (21) are important factors affecting social health (22), the same level of the socio-economic base in active and inactive groups resulted in no significant difference between groups in terms of social health.

The results obtained in the present study regarding the differences between the level of general health and the COVID-19-induced anxiety in active and inactive students are consistent with results of the study by Tofighi et al. (23) who investigated the relationship between physical activity and general health in students of Urmia University of Medical Sciences. These results are also in agreement with the findings of the research by Vedøy et al. (24) who conducted a cross-sectional study on associations between physical activity, mental health, and academic achievement in Norwegian adolescents.

Today, the significant role of physical activity in preventing disorders and promoting mental and physical health is greatly emphasized. In addition, it is considered as a factor that affects self-esteem positively, social adjustment, and cognitive function in healthy individuals (25). As the relationship between body and mind is emphasized in discussions on general health, it seems necessary to recognize the mutual effects of the two on each other in different people and different conditions (26). Mental health is one of the factors assessed when considering health indicators in a society. Adolescence and youth are very important and sensitive stages in the process of human development (27). Physical activity has been recognized as an important tool to improve the level of general health and limited studies have addressed the relationship between the level of physical activity and mental health (28, 29). Public health advocates continue to vigorously promote adequate levels of physical activity in the home to prevent the destructive effects of lifestyles created as a result of protection against COVID-19 or quarantine (30) and to ensure that the restrictions do not lead to the loss of physical activity in people (31).

Inactivity is one of the factors causing many mental disorders, so that several studies confirm that people with mental disorders should be sought among sedentary people (32). In this regard, the results of the present study showed that there was a significant difference between active and inactive students in terms of COVID-19-induced anxiety and general health. Research has shown that exercise can alter the accumulation of monoamine receptors, analgesics, endorphins, and enkephalins. Thus, it can be very effective in positive mood changes. The psychosocial effects of physical activity on depression and anxiety are considerable. It can reduce anxiety and depression in active people compared to their inactive peers by creating opportunities for social interaction and experiencing the feelings of self-efficacy and self-esteem and relieving daily stressors (33). Other possible mechanisms for the anxiolytic effects of exercise are the mediation through the endogenous opioid system that has a significant role in the regulation of mood and emotional responses and the increase in brain-derived neurotrophic factor (BDNF) which is the most abundant neurotrophin in the brain (34). In this regard, we can refer to the study by Gottschlich et al. (32) who examined the relationship between physical activity and the general health of people. The results obtained from the study showed a positive and significant correlation between physical activity and general health. The findings of the present study also showed a significant difference between active and inactive students in terms of general health. These results are consistent with findings of some research that indicate inadequate physical activity and physical weakness in most students of some universities (33). The results of the study by Tehrani et al. (35) also demonstrated that people could develop their psychological and social functions by engaging in appropriate physical activities and participating in exercise programs while enjoying their physical and health benefits.

## Conclusions

Improved general health is necessary for maintaining the desirable social and educational performance of people in a society. Students are prone to losing general health (physical and mental health) due to special conditions of their lives such as being away from family, presence in large and stressful groups, economic problems, the large volume of courses, and so on. Especially during the coronavirus pandemic, when there is a large amount of disturbing and stressful news and the students are anxious about infection (either themselves or their relatives) and the consequences of the disease, the risk of losing mental health is increased in students. Some of the limitations of this study were the lack of access to an equal number of active and inactive students and the precise control of other psychological factors affecting students' general-social health in addition to anxiety. Also, this study could not collect data from all campuses of Farhangian University, only two campuses of Mashhad were chosen for this study. Future research should consider different groups of people to evaluate general-social health in the COVID-19 pandemic.

One of the main tasks of the education system is to help people in a society to enjoy good health conditions. This can be achieved by changing people's lifestyles from a sedentary one to a lifestyle with an appropriate rate of physical activity. Preventing diseases and providing the grounds for general health seem essential, especially for students who are on the front line of the country's education system. Therefore, it is recommended that students enjoy the benefits of an active life with a regular exercise program to reduce their anxiety and improve their physical health.

## Data Availability Statement

The raw data supporting the conclusions of this article will be made available by the authors, without undue reservation.

## Ethics Statement

The studies involving human participants were reviewed and approved by IR.MUMS.REC.1399.445. The patients/participants provided their written informed consent to participate in this study.

## Author Contributions

The author confirms being the sole contributor of this work and has approved it for publication.

## Funding

This work was supported by the Farhangian University, Khorasan Razavi Province, Iran [Grant No. 50000/16783/600].

## Conflict of Interest

The author declares that the research was conducted in the absence of any commercial or financial relationships that could be construed as a potential conflict of interest.

## Publisher's Note

All claims expressed in this article are solely those of the authors and do not necessarily represent those of their affiliated organizations, or those of the publisher, the editors and the reviewers. Any product that may be evaluated in this article, or claim that may be made by its manufacturer, is not guaranteed or endorsed by the publisher.

## References

[1] Freeman S Eykelbosh A. COVID-19 and outdoor safety: Considerations for use of outdoor recreational spaces. National Collaborating Centre for Environmental Health, (2020) 829.

[2] Chen P Mao L Nassis GP Harmer P Ainsworth B Li F. Returning Chinese school-aged children and adolescents to physical activity in the wake of COVID-19: actions and precautions. Sport Health Sci. (2020) 9:103–4. 10.1016/j.jshs.2020.02.001 32325023 PMC7154517

[3] Brooks SK Webster RK Smith LE Woodland L Wessely S Greenberg N. The psychological impact of quarantine and how to reduce it: rapid review of the evidence. Lancet. (2020) 395:912–20. 10.1016/S0140-6736(20)30460-8 32112714 PMC7158942

[4] Eriksson M. Social capital, as a contex for mental health promotion. Social Welfare. (2003) 2:43–53. 10.3402/gha.v4i0.5611 21311607 PMC3036711

[5] Kikuchi S Kitao S Mikoshiba M. Who suffers from the COVID-19 shocks? Labor market heterogeneity and welfare consequences in Japan. J Jpn Int Econ. (2021) 59:101117. 10.1016/j.jjie.2020.101117

[6] Del Turco S Vianello A Ragusa R Caselli C Basta G. COVID-19 and cardiovascular consequences: is the endothelial dysfunction the hardest challenge? Thromb Res. (2020) 196:143–51. 10.1016/j.thromres.2020.08.039 32871306 PMC7451195

[7] Meji MA Dennison MS. Survey on general awareness, mental state and academic difficulties among students due to COVID-19 outbreak in the western regions of Uganda. Heliyon. (2020) 6:e05454. 10.1016/j.heliyon.2020.e05454 33195850 PMC7647392

[8] Butler MJ Barrientos RM. The impact of nutrition on COVID-19 susceptibility and long-term consequences. Brain Behav Immun. (2020) 87:53–54. 10.1016/j.bbi.2020.04.040 32311498 PMC7165103

[9] Chekroud SR Gueorguieva R Zheutlin AB Paulus M Krumholz HM Krystal JH. Association between physical exercise and mental health in 1· 2 million individuals in the USA between 2011 and 2015: a cross-sectional study. Lancet Psychiatry. (2018) 5:739–46. 10.1016/S2215-0366(18)30227-X 30099000

[10] Carek PJ Laibstain SE Carek SM. Exercise for the treatment of depression and anxiety. Int J Psychiatry Med. (2011) 41:15–28. 10.2190/PM.41.1.c 21495519

[11] Nguyen J Brymer E. Nature-based guided imagery as an intervention for state anxiety. Front Psychol. (2018) 9:1858. 10.3389/fpsyg.2018.01858 30333777 PMC6176042

[12] Cao J Wen M Shi Y Wu Y He Q. Investigation of anxiety, depression and influencing factors in patients with new coronavirus pneumonia. J Nursing. (2020) 35:15–7.

[13] Fang BJX Liu YT CX. Reports on self-reporting Inventory (SCL-90) for doctors in recent eleven years and construction of norm: a meta-analysis. Mod Prev Med. (2017) 44:1642–6.

[14] Ripon RK Mim SS Puente AE Hossain S Babor MMH Sohan SA. COVID-19: psychological effects on a COVID-19 quarantined population in Bangladesh. Heliyon. (2020) 6:e05481. 10.1016/j.heliyon.2020.e05481 33200105 PMC7654365

[15] Goldberg DP Gater R. Sartorius N Ustun TB Piccinelli M Gureje O. The validity of two versions of the GHQ in the WHO study of mental illness in general health care. Psychol Med. (1997) 27:191–7. 10.1017/S0033291796004242 9122299

[16] Taghavi M. Assessment of the validity and reliability of the general health questionnaire. J Psychol. (2001) 13:381–9.17478411

[17] Keyes CLM Shapiro AD. Social Well-Being in the United States: A Descriptive Epidemiology. In: Brim OG Ryff CD Kessler RC editors. How Healthy Are We?: A National Study of Well-Being at Midlife. The University of Chicago Press. (2004). p. 350–72.

[18] Sharbatiyan MH. The semantic components reflecting the link between social capital and the rate social health of the benefit of students of Payam Noor University, Mashhad. J Soc Stud Youth. (2012) 2:149–74.

[19] Alipour A Ghadami A Alipour Z Abdollahzadeh H. Preliminary validation of the Corona Disease Anxiety Scale (CDAS) in the Iranian Sample. Q J Health Psychol. (2020) 8:163–75. 10.30473/HPJ.2020.52023.4756

[20] Lesser IA Nienhuis CP. The impact of COVID-19 on physical activity behavior and well-being of Canadians. Int J Environ Res Public Health. (2020) 17:3899. 10.3390/ijerph17113899 32486380 PMC7312579

[21] Chinekesh AHS Mohammadi FS Motlagh ME Eftekhari MB Ardalan G Djalalinia S. Factors affecting social health from young adults' perspective: a qualitative study. Int J Prev Med. (2019) 10:13–8. 10.4103/ijpvm.IJPVM_13_18 31579139 PMC6767806

[22] Ahnquist J Wamala SP Lindstrom M. Social determinants of health–a question of social or economic capital? Interaction effects of socioeconomic factors on health outcomes. Soc Sci Med. (2021) 74:930–9. 10.1016/j.socscimed.2011.11.026 22305807

[23] Tofighi A Babaei S Eloon Kashkuli F Babaei R. The relationship between the amount of physical activity and general health in urmia medical university students. J Nurs. (2014) 12:166–172.

[24] Vedøy IB Anderssen SA Tjomsland HE Skulberg KR Thurston M. Physical activity, mental health and academic achievement: a cross-sectional study of Norwegian adolescents. Ment Health Phys Act. (2020) 18:100322. 10.1016/j.mhpa.2020.100322

[25] Esfahani N. The impact of sport on physical, anxiety, sleep disorder, social function and depression components of mental health in Azzahra University students. Harakat. (2002) 12:75–86.

[26] Aşçi FH. The effects of physical fitness training on trait anxiety and physical self-concept of female university students. Psychol Sport Exerc. (2003) 4:255–64. 10.1016/S1469-0292(02)00009-2

[27] Sharper M. Factors affecting attitudes of students towards sports leisure. J Hosp. (2008) 7:4–14.

[28] Arent SM Landers DM Etnier JL. The effects of exercise on mood in older adults: a meta-analytic review. J Aging Phys Act. (2000) 8:407–30. 10.1123/japa.8.4.40734093345

[29] Bolognini M Plancherel B Nunez R Bettschart W. Assessment of the effects of age at start of puberty on mental health in pre-adolescence: results of a longitudinal study (1989–1991). Rev Epidemiol Sante Publique. (1993) 41:337–45. 8372255

[30] Peluso MA Guerra de Andrade LH. Physical activity and mental health: the association between exercise and mood. Clinics. (2005) 60:61–70. 10.1590/S1807-59322005000100012 15838583

[31] De Moor MH Boomsma DI Stubbe JH Willemsen G de Geus EJ. Testing causality in the association between regular exercise and symptoms of anxiety and depression. Arch Gen Psychiatry. (2008) 65:897–905. 10.1001/archpsyc.65.8.897 18678794

[32] Gottschlich EA Larson K Sisk B Frintner MP. Sleep, physical activity, and general health status: US pediatricians and the general US adult population. Acad Pediatr. (2019) 19:269–77. 10.1016/j.acap.2018.08.002 30103049

[33] Poissonner C-M Iwatsubo Y Cosquer M Salva M-AQ Caillard J-F Veron M. Across-sectional study of the health effects of work schedules on 3212 hospital workers in France: implications for the new french work schedules policy. J Hum Ergol. (2001) 30:387–91. 14564913

[34] Bodnar RJ Klein GE. Endogenous opiates and behavior: 2005. Peptides. (2006) 27:3391–478. 10.1016/j.peptides.2006.07.011 16973239

[35] Tehrani H VSM Fadayevatan R Abusalehi A Esmaeili H. Mental health status and its related factors in elderly people residing in nursing homes of Mashhad. J Health Dev. (2017) 6:171–81.

